# Ergoapiol in Diseases of the Female

**Published:** 1902-08

**Authors:** 


### ABSTRACTS.

### Ergoapiol in Diseases of the Female.

CHARLES H. SHEPARD, M. D., Physician to Lincoln Hospital, Durham, N. C., in an article with the foregoing title, very properly says that a deep and general interest is attached to all knowledge pertaining to the treatment of common diseases of the uterus, and the outcome of this profound and focussed interest is a vast literature. Many former theories in medicine are passing away. New medicines achieve a short-lived success, then pass to obscurity. This is especially so with medicines for gynecological diseases.

All diseases of the womb, of course, have not the same etiology nor the same pathology; therefore they should not have the same treatment. Too often the physician groups these diseases as one and gives a routine treatment. It is not enough to give anodyne medicines for dysmenorrhea, nor is it sufficient to treat alike all forms of dysmenorrhea.

The operation of curetage has a most important place in these conditions, but like other remedial agencies it has its limitations. When we curet the uterus we rid it of a pathologically obnoxious lining membrane and afford a normal membrane the opportunity to be formed.

The healthy woman with normal genitalia menstruates regularly and painlessly once a month from puberty to menopause, except this regularity be interrupted by pregnancy and lactation. Any departure from this rule constitutes an abnormality. Amenorrhea is less frequently met with than dysmenorrhea and irregular menstruation.

The most generally useful medicine in the conditions of amenorrhea, dysmenorrhea, irregular, scanty and fetid menstruation, in my judgment, is a preparation of the Martin H. Smith Company, of New York, known as ergoapiol. In the female ward of the Lincoln Hospital, I have used this medicine extensively, and it has not only rarely failed to benefit or cure, but I know no remedy with which I could replace it.

This medicine is put up as a small capsule, and combined with it are some other valuable hemagogues. What I have to say of this preparation is based entirely upon clinical experience, and a few cases are reported indicating the appropriate use of the drug and its various applicability.

Case I.—Miss W., tubercular history; menstruation very irregular, sometimes three, sometimes five weeks between periods; very painful; scanty. Ergoapiol one capsule four times a day beginning one week before the menstrual period and continued a week after the period. As a result the patient feels better in her general health; her monthly flow has been rendered painless and increased in quantity. Ergoapiol appears to have a tonic action upon the muscular fibers of the womb; not transitory but lasting.

Case II.—Endometritis. Mrs. D. has pain between periods, aggravated at periods. Leucorrhea pronounced; pains in the back; "hot flushes"; vertigo, headache. Patient would not allow an operation; highly sensitive. Several preparations were tried, but none gave relief until ergoapiol was used. Treatment was kept up for ten weeks, when she was symptomatically relieved and called herself well.

Case III.—Mrs. D., widow; aged 33; had three children; youngest ten years of age. Suffered all her menstrual life with pains in the pelvis at each period; had to keep in bed a week or more each month; paroxysms of pain were followed by leucorrhea; no anemia; womb found to be flabby and relaxed; pains extended down thighs posteriorly. Had been treated for many years by various physicians of note, but had received only temporary benefit. Ergoapiol exhibited, one capsule three times a day, and increased at the time of the flow to four a day. After three months of this treatment her menstrual function became regular and she is entirely well.

I could prolong this list with cases that have been entirely relieved by the use of ergoapiol (Smith), but reserve them for further observation and perhaps publication.

